# Follistatin levels and endocrine disorders: A two-sample Mendelian randomization study

**DOI:** 10.1097/MD.0000000000045566

**Published:** 2025-10-31

**Authors:** Wei Zhang, Tianqiang Wu, Xin Zhao, Yidan Ma, Xinyu Han

**Affiliations:** a Department of Critical Care Medicine, Wuhan Jinyintan Hospital, Tongji Medical College of Huazhong University of Science and Technology, Hubei Province, China; b Department of First Clinical Medical College, Heilongjiang University of Chinese Medicine, Harbin, China; c Department of Gynecology, Wenzhou Traditional Chinese Medicine Hospital Affiliated to Zhejiang Chinese Medical University, Wenzhou, China.

**Keywords:** causality, follistatin, Mendelian randomization, osteoporosis, polycystic ovary syndrome, type 2 diabetes

## Abstract

To investigate the causal relationship between follistatin (FST) levels and endocrine diseases such as polycystic ovary syndrome (PCOS), type 2 diabetes (T2DM), obesity, and osteoporosis (OP) using a 2-sample Mendelian randomization (MR) analysis. Instrumental variables closely associated with FST levels were obtained from large-scale genome-wide association study data in the IEU database. Summary-level data for 4 endocrine diseases were sourced from the latest version of the FinnGen database. Our primary method for MR analysis was the inverse-variance weighted (IVW) method, supplemented by the MR-Egger and Weighted Median methods. We conducted a series of sensitivity tests to assess the reliability of our MR results. The IVW analysis revealed a significant causal relationship between elevated levels of FST and both PCOS (odds ratio [OR] = 1.129, 95% confidence interval [CI]: 1.042–1.224, *P* = .003) and T2DM (OR = 1.103, 95% CI: 1.02–1.187, *P* = .01). However, the IVW model did not indicate a causal connection between FST levels and either OP (OR = 1.061, 95% CI: 0.909–1.238, *P* = .452) or obesity (OR = 1.082, 95% CI: 0.983–1.192, *P* = .108). The reverse MR analysis results indicated a causative association between T2DM (OR = 1.047, 95% CI: 1.006–1.089, *P* = .023) and an elevation in FST levels, as well as a causal link between OP (OR = 0.889, 95% CI: 0.804–0.982, *P* = .021) and a reduction in FST levels. There is no direct causality between PCOS (OR = 0.925, 95% CI: 0.778–1.098, *P* = .372), obesity (OR = 1.035, 95% CI: 0.968–1.107, *P* = .312), and FST levels. In addition, our sensitivity tests, which included a pleiotropy test, heterogeneity test, and “leave-one-out” analysis, consistently confirmed the reliability of our results. Genetically predicted high FST levels are causally associated with increased risks of PCOS and T2DM, indicating a potential role in endocrine disease pathogenesis. Moreover, reverse MR analysis revealed a significant causal link between OP and decreased FST levels, suggesting that FST may serve as a promising biomarker or therapeutic target in bone metabolism.

## 1. Introduction

Follistatin (FST) is a member of the transforming growth factor-beta (TGF-β) superfamily. It is a single-chain glycosylated protein primarily synthesized and secreted by the liver. Initially isolated from ovarian follicular fluid, FST can inhibit the activity of follicle-stimulating hormone and regulate follicular development through autocrine or paracrine mechanisms.^[1]^ FST is expressed in nearly all tissues of the human body, and its secretion is regulated by the ratio of glucagon to insulin.^[2–4]^ Within the TGF-β signaling pathway, FST functions as a binding protein and regulatory factor. It participates in regulating various physiological processes, including granulosa cell proliferation, steroid production, insulin secretion, and bone metabolism. FST achieves these regulatory roles by binding to proteins such as activin, myostatin, and bone morphogenetic protein.^[5–7]^

Recent research has suggested that FST may play a role in reproductive endocrine dysfunction, particularly in polycystic ovary syndrome (PCOS). Abnormal levels of FST have been associated with disrupted follicular development and hormonal imbalance in both animal and human studies, implicating FST as a potential contributor to the pathophysiology of PCOS.^[8,9]^ These observations provide a rationale for investigating the causal relationship between genetically predicted FST levels and PCOS in the present study.

In addition to its established role in reproductive endocrine disorders such as PCOS, FST has been increasingly recognized as a potential modulator of metabolic homeostasis. Multiple studies have reported that circulating FST levels are moderately elevated in patients with type 2 diabetes mellitus (T2DM) and are positively correlated with fasting glucose, serum insulin, C-peptide, glycated hemoglobin (HbA1c), and indices of hepatic and adipose tissue insulin resistance (IR).^[10–12]^ FST has also been associated with dyslipidemic traits, including higher serum triglycerides and total cholesterol levels,^[11]^ further suggesting a role in systemic glucose and lipid metabolic regulation.

Although osteoporosis (OP) is traditionally viewed as a skeletal disorder, increasing evidence suggests that it shares common endocrine regulatory pathways with metabolic diseases such as PCOS and T2DM. FST, as an antagonist of activin and myostatin in the TGF-β superfamily, plays a crucial role in musculoskeletal homeostasis. Animal studies have demonstrated that FST-based interventions can enhance bone mass and microarchitecture in osteoporotic models. Clinical data indicate that circulating FST levels are associated with sarcopenia, a condition often coexisting with OP and linked to endocrine dysfunction.^[13,14]^ These findings highlight the potential of FST as a systemic endocrine regulator involved in both metabolic and skeletal disorders. Therefore, we included OP in this study to explore whether genetically predicted FST levels are causally associated with bone health, expanding our understanding of FST’s endocrine relevance.

Mendelian randomization (MR) is an epidemiological research method that uses genetic data as a bridge to investigate causal relationships between specific exposures and outcomes.^[15]^ Traditionally, randomized controlled trials (RCTs) have been considered the gold standard for causal inference; however, they are often costly and complex to execute. MR investigations employ single nucleotide polymorphisms (SNPs) from genome-wide association studies (GWAS) as instrumental variables (IVs), similar to those used in RCTs. This strategy reduces MR study confounding issues by randomly assigning genetic differences during conception. Open datasets of genetic variants and disease information simplify implementation and avoid ethical constraints, making MR studies more viable than RCTs.^[16,17]^ Given the unique advantages of MR, we conducted a 2-sample MR analysis using GWAS summary data from the UK Biobank and Finnish databases. This study aimed to identify potential causal relationships between FST levels and 4 endocrine diseases, thereby contributing new evidence to the field of research.

## 2. Materials and methods

### 2.1. Study design

**Figure 1.**
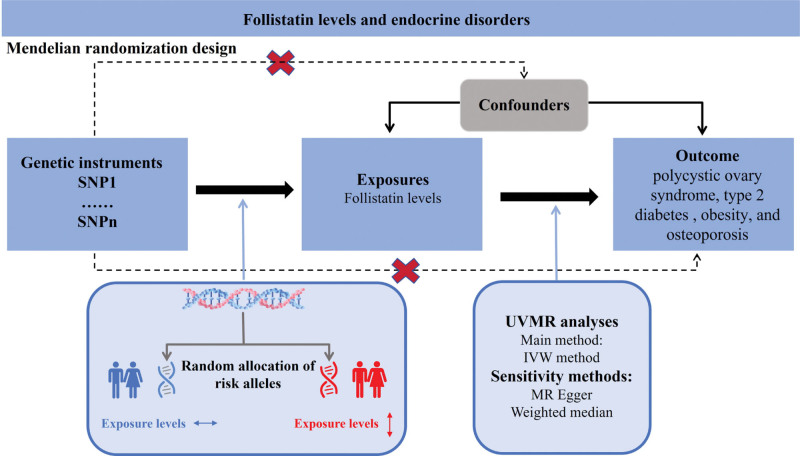
Assumptions of the MR analysis for FST levels and the risk of endocrine disorders. FST = follistatin, MR = Mendelian randomization.

The current study, depicted in Figure 1, employed an MR design, utilizing SNPs as IVs to assess the causal relationship between exposure (FST) and outcomes (PCOS, T2DM, obesity, and OP). In MR studies, the validity of causal estimates hinges on fulfilling 3 critical assumptions: first, the genetic variants must have a robust association with the exposure; second, they should not be associated with any potential confounders of the exposure–outcome relationship; and third, the variants should not have a separate impact on the outcome, apart from their correlation with the exposure.^[18]^ Our MR research adopted ethically approved and informed consent-based publically published studies and datasets. No extra ethical declarations or consents were needed for this analysis.

### 2.2. Data sources

The GWAS summary statistics for FST were obtained from a comprehensive GWAS study of 90 cardiovascular-related proteins across 15 studies published by Folkersen et al^[19]^ in 2020. The study encompassed a total of 21,758 individuals in the European population. It covered a total of 13,022,208 SNPs. In order to prevent inflated type 1 errors brought about by sample overlap, the summary-level data for the following endocrine disorders: PCOS (34,388 cases and 195,922 controls), T2DM (65,085 cases and 335,112 controls), obesity (23,971 cases and 388,084 controls), and OP (8017 cases and 391,037 controls) were acquired from the FinnGen consortium. The cases of 4 endocrine diseases were identified through hospital records and primary healthcare records in Finland using the International Classification of Diseases (ICD-9 and ICD-10). Table 1 presents a concise overview of all datasets incorporated in this investigation.

**Table 1:** Details of studies included in Mendelian randomization (MR) analyses.

Disease or trait	Sample size(cases/controls)	GWAS ID	Ancestry	Year	PMID or consortium
FST	21,758	ebi-a-GCST90012080	European	2020	33067605
PCOS	34,388/195,922	-	European	2023	FinnGen consortium
T2DM	65,085/335,112	-	European	2023	FinnGen consortium
Obesity	23,971/388,084	-	European	2023	FinnGen consortium
Osteoporosis	8017/391,037	-	European	2023	FinnGen consortium

FST = follistatin, GWAS = genome-wide association study, PCOS = polycystic ovary syndrome, PMID = PubMed identifier, T2DM = type 2 diabetes.

### 2.3. Selection and evaluation of IV

To fulfill the 3 critical assumptions of MR analysis, we implemented a specific procedure for selecting the IVs. We extracted SNPs strongly related to FST at the significance level of *P* < 5 × 10^−6^ due to the limited number of available SNPs when adopting a cutoff threshold (*P* < 5 × 10 ^-8^) to identify SNPs predictive of FST. To mitigate the impact of linkage disequilibrium among the SNPs, we established a stringent criterion (*r*^2^ < 0.001 and a clumping distance of 10,000 kb), ensuring that the selected IVs were conditionally independent. Only SNPs with the lowest *P*-values were retained.^[20]^ Furthermore, the potential pleiotropic effects were controlled by extracting the secondary phenotype of each SNP from LDlink (https://ldlink.nih.gov/?tab=ldtrait).^[21]^ SNPs that matched the observed phenotypes were removed in subsequent analysis. Ultimately, we extracted exposure IVs from the outcome data and conducted data harmonization to exclude SNPs with inconsistent alleles of both exposure and outcome data.

The robustness of IVs was evaluated employing variance (*R*^2^) and F-statistic to mitigate the influence of weak instrument bias. The formula to calculate the F-statistic for each SNP is F = *R*^2^/(1 − *R*^2^) [(N − *K* − 1)/*K*], where *N* represents the sample size, *K* denotes the total number of SNPs selected for MR analysis, and *R*^2^ reflects the overall proportion of phenotypic differences explained by all the SNPs in our MR model.^[22]^ The *R*^2^ for each SNP was calculated utilizing the following formula: *R*^2^ = 2 * EAF * (1 − EAF) * β^2^, where β is the β coefficient for effect size and EAF is the effect allele frequency for each SNP.^[23]^ An F-statistic exceeding 10 was considered significant for the association between the IVs and exposure, ensuring that the results were not affected by weak instrument bias.^[24]^ Statistical power for each outcome was determined using the online tool available at https://shiny.cnsgenomics.com/mRnd/.^[25]^ We recommend achieving a sufficient power of at least 80% for robust results.

### 2.4. Statistical analysis

To assess the genetic causal effects, various methodologies, including inverse-variance weighted (IVW), MR-Egger, and weighted median, were implemented. These methods yielded reliable evidence under different circumstances, with IVW being the primary result.^[26]^ The IVW technique is an expansion of the Wald ratio estimator that utilizes meta-analytic principles. It aims to provide an unbiased estimation in an optimal scenario where all the included SNPs are assumed to be legitimate IVs without any horizontal pleiotropy or heterogeneity.^[27]^ MR-Egger allows specific SNPs to impact the result through mechanisms other than exposure, which can provide a dependable and impartial estimate, even when not all SNPs are valid. Furthermore, the MR-Egger intercept can identify and correct for pleiotropy.^[26]^ Although up to 50% of the data utilized in the study consists of invalid IVs, the weighted median method can still generate dependable estimates of the causal effects.^[28]^

In this study, a series of sensitivity analyses were conducted to confirm the stability and reproducibility of the MR results. Cochran Q test was employed to evaluate heterogeneity among SNPs, with a *P*-value exceeding .05 signifying no significant heterogeneity. Additionally, the MR-Egger intercept approach was utilized to estimate the degree of horizontal pleiotropy attributable to IVs. A leave-one-out analysis was also performed to determine whether the MR findings were influenced by any specific SNP. Furthermore, the MR-PRESSO method was applied to detect potential outlier SNPs. To adjust for multiple comparisons, the *P*-value was corrected using the Bonferroni method. The association between FST and 4 endocrine disorders was deemed statistically significant at a 2-sided *P*-value of <.0125 (α = 0.05/4 outcomes) and suggestive when the *P*-value was under .05. MR analyses were performed using the TwoSampleMR (version 0.5.6) package in R (version 4.3.1).

## 3. Result

### 3.1. Genetic instruments

In this study, we identified 5, 6, 9, and 6 SNPs as IVs for FST to evaluate its associations with PCOS, T2DM, obesity, and OP, respectively, as detailed in Supplementary File (Tables S1–S4, Supplemental Digital Content, https://links.lww.com/MD/Q491). The F-statistics for these genetic variants were all above the critical value of 10, suggesting a minimal risk of weak instrumental bias. The statistical power for PCOS and T2DM exceeded or approached 80%, affirming the reliability of these results. However, the statistical power for other phenotypes fell below 80%, raising the possibility of false negatives. Table 2 comprehensively presents the strength and statistical power of the selected IVs. Furthermore, in the MR analysis of endocrine diseases on FST, we chose 4, 130, 30, and 6 SNPs associated with PCOS, T2DM, obesity, and OP, respectively, as detailed in Supplementary File (Tables S5–S8, Supplemental Digital Content, https://links.lww.com/MD/Q491). The F-statistics for these SNPs also surpassed the threshold of 10, indicating a low risk of weak instrumental bias.

**Table 2:** The results of the selected IVs strength and statistical power.

Exposures	Outcomes	*R*^2^ for TL (Total)	F for TL (Total)	Power
FST	PCOS	0.024	534.984	0.92
	T2DM	0.014	302.935	0.79
	Obesity	0.017	376.248	0.37
	Osteoporosis	0.023	512.168	0.13

FST = follistatin, IVs = instrumental variables, PCOS = polycystic ovary syndrome, T2DM = type 2 diabetes.

### 3.2. Estimated causal effect of FST on endocrine diseases

**Figure 2.**
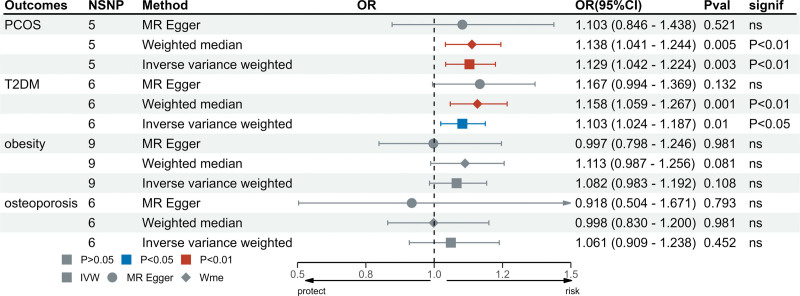
The causal association of genetically predicted FST levels and the risk of endocrine disorders. FST = follistatin.

**Figure 3.**
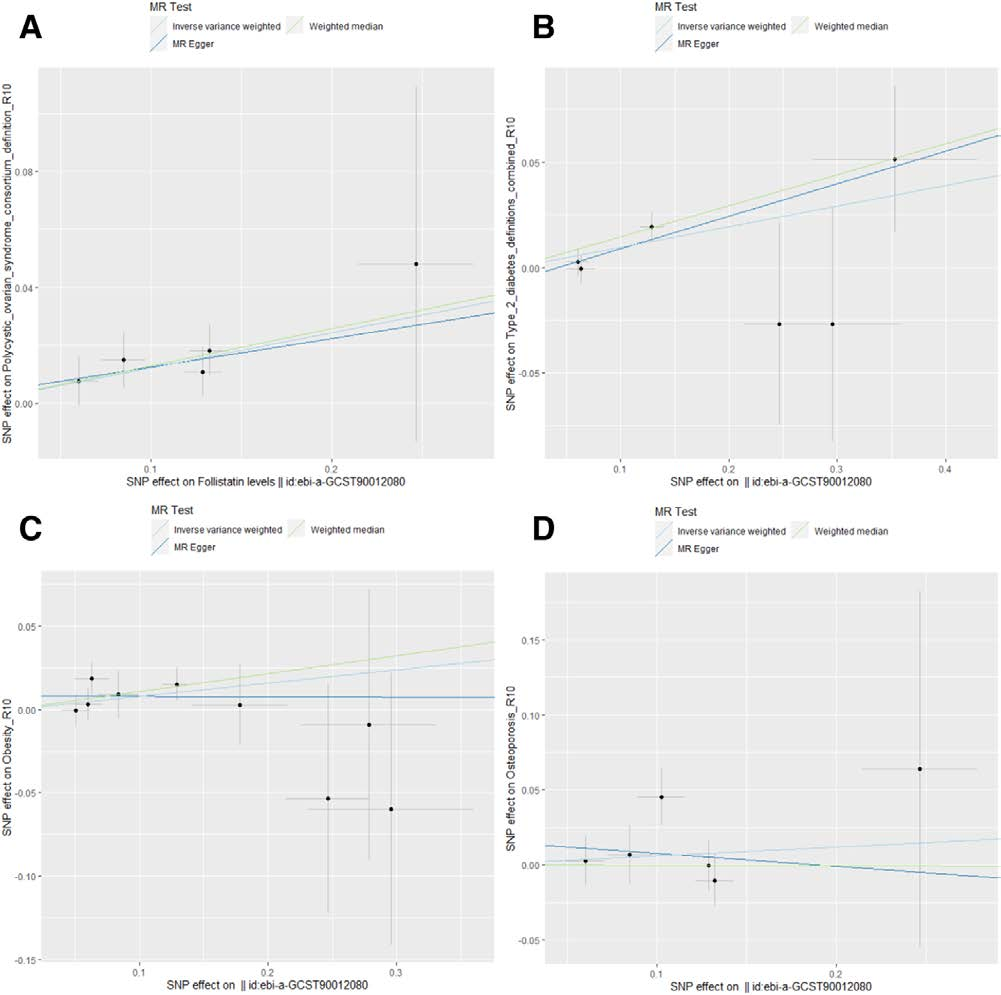
Scatter plots for MR analyses of the correlation between FST levels and endocrine disorders. (A) PCOS; (B) T2DM; (C) obesity; (D) osteoporosis. FST = follistatin, MR = Mendelian randomization, PCOS = polycystic ovary syndrome, T2DM = type 2 diabetes.

After applying the Bonferroni correction, the IVW analysis revealed a significant causal relationship between elevated levels of FST and both PCOS (odds ratio [OR] = 1.13, 95% confidence interval [CI]: 1.04–1.22, *P* = .003) and T2DM (OR = 1.10, 95% CI: 1.02–1.19, *P* = .01). These results aligned with those from the Weighted median model. Conversely, the IVW model did not indicate a causal connection between FST levels and either OP (OR = 1.06, 95% CI: 0.91–1.24, *P* = .451) or obesity (OR = 1.08, 95% CI: 0.98–1.19, *P* = .108). Similar conclusions were drawn from the other 2 statistical models. Figure 2 demonstrates the causal link between genetic predictors of FST and the risk of endocrine diseases. The scatter plots of the associations between FST and 4 types of endocrine disorders are shown in Figure 3.

### 3.3. Estimated causal effect of endocrine diseases on FST

**Figure 4.**
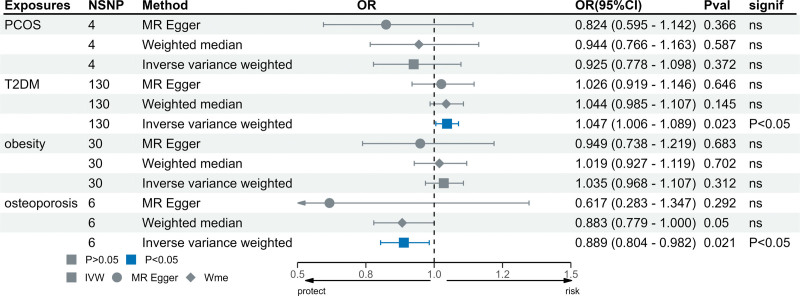
The causal association of genetically predicted endocrine disorders and FST levels. FST = follistatin.

**Figure 5.**
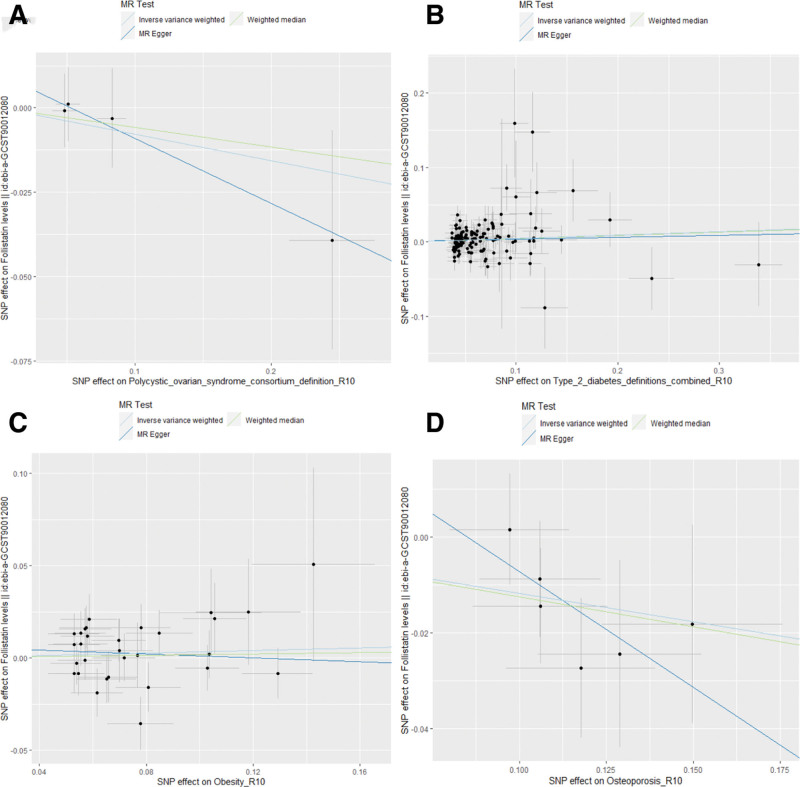
Scatter plots for MR analyses of the correlation between endocrine disorders and FST levels. (A) PCOS; (B) T2DM; (C) obesity; (D) osteoporosis. FST = follistatin, MR = Mendelian randomization, PCOS = polycystic ovary syndrome, T2DM = type 2 diabetes.

The reverse MR analysis results indicated a causative association between T2DM (OR = 1.047, 95% CI: 1.006–1.089, *P* = .023) and an elevation in FST levels, as well as a causal link between OP (OR = 0.889, 95% CI: 0.804–0.982, *P* = .021) and a reduction in FST levels. There is no direct causality between PCOS (OR = 0.925, 95% CI: 0.778–1.098, *P* = .372), obesity (OR = 1.035, 95% CI: 0.968–1.107, *P* = .312), and FST levels. Figure 4 illustrates the causal relationship between genetic predictors of endocrine diseases and FST levels. The scatter plots of the associations between 4 types of endocrine disorders and FST are shown in Figure 5.

### 3.4. Sensitivity analysis results

Cochran Q test showed no evidence of heterogeneity, and the MR-Egger intercept test found no indications of horizontal pleiotropy in the MR analyses. Additionally, the MR-PRESSO analysis identified no outliers among the SNPs. Tables 3 and 4 provide detailed results from the sensitivity analyses. The “leave-one-out” plots in our study confirm the robustness of our findings, suggesting a negligible impact of any individual SNP on the causal estimates (see Supplementary File, Figures S1–S8, Supplemental Digital Content, https://links.lww.com/MD/Q490).

**Table 3:** Heterogeneity, horizontal pleiotropy, and MR-PRESSO tests of the associations between FST levels and endocrine disorders.

Outcomes	Pleiotropy test			Heterogeneity test						MR-PRESSO
	MR-Egger			MR-Egger			Inverse-variance weighted			Global Test
	Intercept	SE	*P*	Q-value	Q-df	Q-*P*val	Q-value	Q-df	Q-*P*val	*P*-value
PCOS	0.002	0.015	.869	0.665	3	.881	0.698	4	.952	.934
T2DM	-0.006	0.008	.478	4.046	4	.399	4.664	5	.458	.426
Obesity	0.008	0.01	.450	4.263	7	.749	4.901	8	.768	.802
Osteoporosis	0.016	0.033	.647	5.427	4	.246	5.759	5	.330	.353

MR-PRESSO = Mendelian randomization pleiotropy residual sum and outlier, PCOS = polycystic ovary syndrome, Q-value = the statistics of Cochran Q test, SE = standard error, T2DM = type 2 diabetes.

**Table 4:** Heterogeneity, horizontal pleiotropy, and MR-PRESSO tests of the associations between endocrine disorders and FST levels.

Exposures	Pleiotropy test			Heterogeneity test						MR-PRESSO
	MR-Egger			MR-Egger			Inverse-variance weighted			Global test
	Intercept	SE	*P*	Q-value	Q-df	Q-*P*val	Q-value	Q-df	Q-*P*val	*P*-value
PCOS	0.01	0.013	.502	0.067	2	.967	0.725	3	.867	.832
T2DM	0.001	0.003	.709	132.05	128	.385	132.194	129	.406	.409
Obesity	0.006	0.009	.484	30.245	28	.351	30.788	29	.375	.414
Osteoporosis	0.041	0.045	.408	1.60	4	.809	2.453	5	.784	.801

MR-PRESSO = Mendelian randomization pleiotropy residual sum and outlier, Q-value = the statistics of Cochran Q test, PCOS = polycystic ovary syndrome, T2DM = type 2 diabetes, SE = standard error.

## 4. Discussion

To the best of our knowledge, this study is the first to investigate the causal relationships between FST levels and common endocrine diseases, specifically, PCOS, T2DM, obesity, and OP, using MR analysis of GWAS datasets. Our findings indicate that high FST levels are a risk factor for the development of polycystic ovary syndrome and type 2 diabetes. However, there is no causal relationship between FST levels and the development of obesity or OP. Reverse MR analysis revealed a causal relationship between T2DM and increased levels of FST, while a correlation was observed between OP and decreased levels of FST. No causal relationship was observed between PCOS, obesity, and FST levels. Multiple sensitivity analyses have consistently confirmed the reliability of our results. These findings are significant for understanding the pathogenesis of these 4 endocrine diseases and may aid in the development of potential prevention and treatment strategies.

PCOS is a common heterogeneous endocrine disorder affecting ~4% to 21% of women of childbearing age worldwide.^[29]^ Besides infrequent ovulation and infertility, among other reproductive issues, PCOS is also linked to IR, T2DM, liver steatosis, and other metabolic disorders.^[30]^ Previous researches have examined the relationship between FST and PCOS. Multiple studies have reported a significant increase in serum FST concentrations in women with PCOS compared with healthy control groups, independent of body mass index (BMI).^[31,32]^ However, 1 study reported that nonobese adolescent girls with PCOS exhibited similar serum FST concentrations compared with healthy girls matched for age and BMI. These discrepancies may be attributed to differences in sample size and age characteristics, as the study focused exclusively on adolescent patients with PCOS.^[33]^ Additionally, variations in biological sample types may also impact the results. Multiple studies have reported that serum FST concentrations are significantly elevated in women with PCOS compared with healthy controls,^[34,35]^ whereas 1 study found no significant difference in FST levels within follicular fluid between PCOS and non-PCOS individuals.^[36]^ This suggests that serum FST may better reflect systemic or metabolic aspects of the disease, while follicular fluid FST levels could be more closely tied to local ovarian microenvironments. Such discrepancies underscore the complexity of FST biology and emphasize the importance of considering sample context when interpreting results.

Our research findings indicate that elevated levels of FST may contribute to the development of PCOS. Existing studies suggest a postulated association between FST and PCOS, which is believed to involve the following pathways. First, FST can disrupt follicle-stimulating hormone secretion by inhibiting activin activity, which hampers follicular development and increases the production of ovarian androgens, both key pathological elements of PCOS.^[32]^ Furthermore, an inverse relationship exists between FST levels and adiponectin, a protein that enhances the body’s response to insulin,^[37]^ and a positive relationship with blood insulin levels and the accumulation of fat in abnormal locations, specifically in the liver. Excessive amounts of FST can result in IR and central obesity, which may give rise to the onset of PCOS.^[33,38]^ Notably, while our forward MR analysis indicated a significant causal effect of elevated FST levels on the risk of PCOS, the reverse MR analysis did not support a causal influence of genetically predicted PCOS on circulating FST levels. This directional asymmetry may be attributed to several factors. First, PCOS is a clinically and genetically heterogeneous disorder encompassing distinct phenotypes characterized by hyperandrogenism, ovulatory dysfunction, or metabolic abnormalities. Such heterogeneity may dilute potential downstream regulatory effects on FST expression, reducing the likelihood of detecting a reverse causal association. Second, FST is a glycoprotein primarily secreted by the liver and functions as an upstream regulator within the TGF-β signaling cascade. Its role in modulating activin and other intraovarian pathways suggests that FST contributes to PCOS pathogenesis rather than being a downstream response to the disease. Moreover, the absence of reverse causality may be explained by physiological context or compensatory mechanisms, such as hormonal feedback regulation or metabolic adaptation, which can influence FST levels in a nonlinear and individual-specific manner. Collectively, these findings support the interpretation that FST is more likely to act as a causal upstream factor in PCOS development rather than serving as a downstream biomarker of the disorder.

T2DM is a chronic, noninfectious disease characterized primarily by hyperglycemia, resulting from a combination of genetic and environmental factors. Dysfunction of β-cells can lead to insufficient insulin secretion, or an impaired insulin response may occur due to IR. T2DM also affects other peripheral tissues and leads to various complications.^[39]^ The 19-year cohort study conducted by Wu et al demonstrated a correlation between elevated circulating levels of FST and a higher likelihood of T2DM, independent of other identified risk factors.^[40]^ Consistent with this, our research also indicates that high levels of FST can increase the risk of developing T2DM. Animal studies have demonstrated that in obese mice fed a diet high in sugar and fat, FST can exacerbate IR in white adipose tissue. Inhibiting FST expression in these models has been shown to restore glucose tolerance.^[3]^ Moreover, in obese patients with diabetes undergoing gastric bypass surgery, decreases in serum FST and HbA1c levels have been observed concurrently.^[41]^ This implies that FST may act as a mediator in the development of T2DM.

Our reverse MR analysis reveals a causal relationship between T2DM and the increase in FST levels, corroborating findings from previous observational studies.^[11]^ However, the significance of increased FST levels in patients with T2DM remains unclear. It is uncertain whether elevated FST levels are a component of metabolic disorders contributing to the development of T2DM or if they represent a compensatory response to hyperglycemia. On the one hand, in mouse models, it has been observed that knocking out a subtype of FST, FST 315, can lead to enhanced insulin signaling in the liver.^[42]^ On the other hand, Davey et al noted that intravascular delivery of the FST gene as an intervention can improve glucose metabolism in diabetic mice. This treatment effectively reduced HbA1c levels to those comparable to those of healthy subjects.^[43]^ Likewise, the excessive production of FST in skeletal muscle has been demonstrated to enhance glucose absorption, along with improving glucose tolerance in mouse models.^[7]^ The presence of different FST subtypes and their varying roles across different tissues may lead to inconsistent findings in research. Therefore, further studies are necessary to clarify the specific functions of FST in the development and management of T2DM.

Obesity is a condition characterized by an abnormal accumulation or distribution of body fat, which hurts health due to metabolic dysfunction. It is commonly related to endocrine disorders such as T2DM and PCOS.^[44]^ Therefore, it is warranted to explore whether obesity influences FST levels. The existing data on the relationship between obesity and changes in circulating FST levels are contradictory. Li et al’s study observed that the expression of follistatin-like 3 (FSTL3) in adipose tissue from overweight or obese individuals was higher compared with that in individuals of normal weight. Additionally, serum FSTL3 levels were positively correlated with BMI, waist circumference, fat mass, and body fat percentage.^[45]^ Perakakis et al discovered that FST levels in morbidly obese patients, both with and without T2DM, significantly decreased following weight loss surgery.^[41]^ Similarly, Wiewiora et al also confirmed that in morbidly obese individuals without diabetes, there was a drop in circulating FST concentrations 1 year after undergoing weight loss surgery.^[46]^

Nevertheless, certain studies have indicated that weight loss surgery can substantially elevate FST concentration 1 year after the procedure in morbidly obese patients who do not have glucose metabolic issues.^[7,47]^ The contradictory results make it difficult to draw reliable conclusions about the effects of FST concentrations on obesity. Besides, numerous studies have shown that there is no substantial correlation between the levels of follistatin in the bloodstream and obesity.^[11,35]^ Sylow et al highlighted that the presence of obesity alone, in the absence of IR, does not affect FST levels.^[10]^ In line with these findings, our study does not provide genetic evidence for a causal relationship between obesity and FST levels. These results suggest that obesity may not have a direct impact on FST levels in the bloodstream. Even so, further investigation is required to validate these findings.

OP is a chronic, progressive systemic skeletal disease characterized by a reduction in bone density and an increased susceptibility to fractures.^[48]^ FST is believed to regulate muscle and bone mass by inhibiting activin and myostatin in bone cells.^[49]^ Animal studies have shown that decreased levels of FST are associated with reductions in bone mineral density, bone volume, and the number and thickness of trabeculae in mice.^[50]^ There are relatively few clinical studies examining the effects of FST on bone tissue, and the results across different populations have been contradictory. One clinical study, after adjusting for age, BMI, and 25-hydroxyvitamin D levels, found no significant differences in the activins-follistatins-inhibins hormonal system between postmenopausal women with hip fractures and those with osteoarthritis.^[51]^ Another study investigated the impact of muscle-related hormones on bone metabolism in obese women. The results indicated that, compared with a normal-weight control group (n = 40), obese women (n = 139) exhibited higher levels of areal bone mineral density (aBMD), muscle growth inhibitors, and FST. However, further analysis revealed that the effect of weight on bone density was not mediated by FST.^[52]^ A recent study revealed that patients with anorexia nervosa have higher levels of myokines, such as FST and irisin, compared with the control group. However, these elevated levels are not linked to a decrease in bone density or alterations in bone turnover when accounting for other well-established factors related to the disease, such as estrogen deficiency.^[53]^ Our MR study results align with the prior clinical trials, indicating that there is no substantial causal link between FST and OP. This means that the direct influence of FST on OP is somewhat limited compared with other risk factors. Interestingly, the reverse MR analysis results demonstrated a causal link between OP and reduced FST levels. Several clinical trials have suggested that FST may have a preventive effect on OP. Studies involving women before and after menopause have shown that decreased levels of inhibin B and increased ratios of activin to FST contribute to the occurrence of OP, regardless of age and BMI.^[51]^ A separate investigation conducted on teenage girls between the ages of 14 and 18 revealed a noteworthy positive relationship between the level of FST in the blood and the total amount of minerals in the bones (*R* = 0.33), the overall density of the bones (*R* = 0.23), and the density of the bones in the lumbar area (*R* = 0.29).^[54]^ The implications of reduced FST levels in individuals with OP are not yet fully understood. The significance of decreasing FST in decreased bone density is unknown. Additional investigation is required to ascertain whether FST could serve as a viable therapeutic target for enhancing bone density.

Previous researches have indicated a potential link between FST and various hormonal diseases. However, the findings remain disputed, and there is a lack of genetic evidence to support them. Establishing a clear causal link between FST and endocrine diseases is challenging due to the presence of factors that can confound the results. To address this challenge, we employed the MR technique, utilizing genetic variation as IVs. This strategy provides numerous benefits: it efficiently reduces the influence of any genetic variants that are often linked to confounding variables in epidemiological investigations. Moreover, the use of a substantial sample size drawn from the most recent Finnish database enhances the statistical reliability of our investigation. Ultimately, we conducted an extensive sensitivity analysis to ensure the reliability of our research results.

Nevertheless, this study has multiple limitations. Due to the dependence on aggregated data from the GWAS database, it is not feasible to assess nonlinear relationships between FST levels and endocrine illnesses. Furthermore, the lack of access to stratified data on FST levels and subtypes in different organizations hinders a thorough and detailed examination of the correlation between FST and endocrine illnesses. In addition, the overrepresentation of individuals of European descent reduces the potential bias caused by population stratification, but it also restricts the applicability of our results to other ethnic groups. Despite the *F*-statistic indicating a robust IV, specific phenotypes have insufficient statistical power (below 80%), which can result in false negatives.

## 5. Conclusion

Genetic variables that result in elevated levels of FST are linked to a higher likelihood of developing specific endocrine disorders, indicating that FST plays a crucial role in the development of conditions such as PCOS and T2DM. This suggests that FST has the potential to be a promising biomarker for targeted therapy. Subsequent investigations should examine interventions designed to control FST levels in order to avoid or mitigate these disorders. These findings underscore the importance of conducting further research to elucidate potential molecular pathways. Furthermore, the exact meaning of reduced FST levels in individuals with OP is yet uncertain. Further investigation is required to determine the potential of FST as a feasible therapeutic target for managing poor bone density.

## Acknowledgments

We would like to thank the researchers and study participants for their contributions.

## Author contributions

**Conceptualization:** Wei Zhang.

**Data curation:** Xinyu Han.

**Formal analysis:** Wei Zhang, Xinyu Han.

**Funding acquisition:** Xinyu Han.

**Methodology:** Tianqiang Wu.

**Validation:** Yidan Ma.

**Writing – original draft:** Wei Zhang, Xin Zhao, Yidan Ma.

**Writing – review & editing:** Tianqiang Wu, Xin Zhao, Yidan Ma.

## Supplementary Material




