# Supplementary material for: Follistatin levels and endocrine disorders: A two-sample Mendelian randomization study
Source: Medicine (Baltimore). 2025 Oct 31;104(44):e45566. doi: 10.1097/MD.0000000000045566 (PMC12582763; doi:10.1097/MD.0000000000045566)
Supplement: Supplementary file 1 [file medi-104-e45566-s001.docx]

**Supplementary Table 1.** The summary information for instrumental variables in FST and PCOS.

| **Sort** | **chr** | **pos** | **SNP** | **effect_allele** | **other_allele** | **beta** | **se** | **eaf** | ***R^2^*** | ***F*** |
| --- | --- | --- | --- | --- | --- | --- | --- | --- | --- | --- |
| 1 | 9 | 92228559 | rs10908903 | G | T | 0.060 | 0.011 | 0.4587 | 0.002 | 30.601 |
| 2 | 2 | 27730940 | rs1260326 | T | C | 0.132 | 0.011 | 0.3976 | 0.008 | 155.764 |
| 3 | 15 | 43726625 | rs150844304 | C | A | 0.247 | 0.032 | 0.0294 | 0.003 | 59.011 |
| 4 | 5 | 53327571 | rs31226 | T | C | 0.129 | 0.011 | 0.4021 | 0.008 | 145.110 |
| 5 | 12 | 57791833 | rs7974833 | C | T | 0.085 | 0.012 | 0.235 | 0.003 | 47.639 |

**Supplementary Table 2.** The summary information for instrumental variables in FST and T2DM.

| **Sort** | **chr** | **pos** | **SNP** | **effect_allele** | **other_allele** | **beta** | **se** | **pval** | ***R^2^*** | ***F*** |
| --- | --- | --- | --- | --- | --- | --- | --- | --- | --- | --- |
| 1 | 9 | 92228559 | rs10908903 | G | T | 0.060 | 0.011 | 2.76E-08 | 0.001 | 30.601 |
| 2 | 8 | 6431959 | rs117993236 | C | T | 0.296 | 0.065 | 4.72E-06 | 0.001 | 20.936 |
| 3 | 15 | 87106777 | rs147341928 | A | G | 0.063 | 0.013 | 1.80E-06 | 0.001 | 22.849 |
| 4 | 11 | 120493965 | rs149630710 | G | A | 0.353 | 0.076 | 3.52E-06 | 0.001 | 21.495 |
| 5 | 15 | 43726625 | rs150844304 | C | A | 0.247 | 0.032 | 1.46E-14 | 0.003 | 59.011 |
| 6 | 5 | 53327571 | rs31226 | T | C | 0.129 | 0.011 | 1.56E-33 | 0.007 | 145.11 |

**Supplementary Table 3.** The summary information for instrumental variables in FST and obesity.

| **Sort** | **chr** | **pos** | **SNP** | **effect_allele** | **other_allele** | **beta** | **se** | **pval** | ***R^2^*** | ***F*** |
| --- | --- | --- | --- | --- | --- | --- | --- | --- | --- | --- |
| 1 | 9 | 92228559 | rs10908903 | G | T | 0.0603 | 0.011 | 2.76E-08 | 0.001 | 30.601 |
| 2 | 8 | 6431959 | rs117993236 | C | T | 0.2956 | 0.065 | 4.72E-06 | 0.001 | 20.936 |
| 3 | 6 | 127905283 | rs141142667 | G | A | 0.2788 | 0.053 | 1.16E-07 | 0.001 | 28.091 |
| 4 | 15 | 87106777 | rs147341928 | A | G | 0.0631 | 0.013 | 1.80E-06 | 0.001 | 22.849 |
| 5 | 15 | 43726625 | rs150844304 | C | A | 0.2466 | 0.032 | 1.46E-14 | 0.003 | 59.011 |
| 6 | 9 | 93716910 | rs188442 | C | T | 0.0508 | 0.011 | 2.51E-06 | 0.001 | 22.123 |
| 7 | 5 | 53327571 | rs31226 | T | C | 0.1289 | 0.011 | 1.56E-33 | 0.007 | 145.11 |
| 8 | 7 | 73033559 | rs34062580 | G | A | 0.0839 | 0.016 | 1.12E-07 | 0.001 | 28.195 |
| 9 | 9 | 91998215 | rs78715695 | T | C | 0.178 | 0.037 | 1.60E-06 | 0.001 | 23.017 |

**Supplementary Table 4.** The summary information for instrumental variables in FST and osteoporosis.

| **Sort** | **chr** | **pos** | **SNP** | **effect_allele** | **other_allele** | **beta** | **se** | **pval** | ***R^2^*** | ***F*** |
| --- | --- | --- | --- | --- | --- | --- | --- | --- | --- | --- |
| 1 | 9 | 92228559 | rs10908903 | G | T | 0.0603 | 0.011 | 2.76E-08 | 0.001 | 30.601 |
| 2 | 2 | 27730940 | rs1260326 | T | C | 0.1323 | 0.011 | 9.58E-36 | 0.007 | 155.764 |
| 3 | 15 | 43726625 | rs150844304 | C | A | 0.2466 | 0.032 | 1.46E-14 | 0.003 | 59.011 |
| 4 | 5 | 53327571 | rs31226 | T | C | 0.1289 | 0.011 | 1.56E-33 | 0.007 | 145.11 |
| 5 | 5 | 52774510 | rs62370480 | G | A | 0.1025 | 0.013 | 2.48E-15 | 0.003 | 62.161 |
| 6 | 12 | 57791833 | rs7974833 | C | T | 0.0849 | 0.012 | 5.61E-12 | 0.002 | 47.639 |

**Supplementary Table 5.** The summary information for instrumental variables in PCOS and FST.

| **Sort** | **chr** | **pos** | **SNP** | **effect_allele** | **other_allele** | **beta** | **se** | **pval** | ***R^2^*** | ***F*** |
| --- | --- | --- | --- | --- | --- | --- | --- | --- | --- | --- |
| 1 | 19 | 49519466 | rs1056917 | A | G | 0.051 | 0.009 | 5.08E-09 | 0.0001 | 34.158 |
| 2 | 11 | 30319433 | rs12271300 | G | A | 0.083 | 0.011 | 4.04E-14 | 0.0002 | 57.146 |
| 3 | 2 | 21069965 | rs12620467 | G | A | 0.048 | 0.009 | 4.35E-08 | 0.0001 | 29.985 |
| 4 | 1 | 169519049 | rs6025 | C | T | 0.245 | 0.031 | 7.78E-15 | 0.0003 | 60.389 |

**Supplementary Table 6.** The summary information for instrumental variables in T2DM and FST.

| **Sort** | **chr** | **pos** | **SNP** | **effect_allele** | **other_allele** | **beta** | **se** | **pval** | ***R^2^*** | ***F*** |
| --- | --- | --- | --- | --- | --- | --- | --- | --- | --- | --- |
| 1 | 2 | 60073321 | rs10084393 | C | A | 0.056 | 0.008 | 1.20E-12 | 0.0001 | 50.484 |
| 2 | 19 | 46157004 | rs10408179 | T | C | 0.06 | 0.007 | 1.04E-19 | 0.0002 | 82.525 |
| 3 | 4 | 6304087 | rs1046316 | G | A | 0.064 | 0.007 | 5.05E-21 | 0.0002 | 88.511 |
| 4 | 12 | 27959201 | rs10466811 | G | A | 0.056 | 0.008 | 8.42E-13 | 0.0001 | 51.181 |
| 5 | 7 | 103567874 | rs10487174 | C | T | 0.037 | 0.007 | 2.21E-08 | 0.0001 | 31.3 |
| 6 | 8 | 41515993 | rs10504042 | A | G | 0.059 | 0.007 | 2.95E-19 | 0.0002 | 80.471 |
| 7 | 11 | 2195267 | rs10770143 | C | T | 0.068 | 0.007 | 5.18E-23 | 0.0002 | 97.577 |
| 8 | 17 | 46197755 | rs10775406 | G | A | 0.047 | 0.007 | 3.59E-10 | 0.0001 | 39.325 |
| 9 | 10 | 94460650 | rs10882099 | T | C | 0.067 | 0.007 | 1.22E-24 | 0.0003 | 105.002 |
| 10 | 9 | 22132729 | rs10965247 | A | G | 0.113 | 0.009 | 5.98E-34 | 0.0004 | 147.539 |
| 11 | 9 | 4291928 | rs10974438 | C | A | 0.044 | 0.007 | 6.80E-11 | 0.0001 | 42.575 |
| 12 | 9 | 119145200 | rs10983127 | T | C | 0.048 | 0.008 | 1.44E-08 | 0.0001 | 32.134 |
| 13 | 16 | 23908271 | rs11074585 | A | G | 0.056 | 0.01 | 2.10E-08 | 0.0001 | 31.396 |
| 14 | 12 | 50248052 | rs11169182 | T | C | 0.041 | 0.007 | 4.02E-10 | 0.0001 | 39.104 |
| 15 | 8 | 19526176 | rs111716216 | C | T | 0.046 | 0.008 | 2.42E-08 | 0.0001 | 31.122 |
| 16 | 19 | 1154395 | rs112016043 | G | T | 0.12 | 0.02 | 3.04E-09 | 0.0001 | 35.16 |
| 17 | 12 | 4271088 | rs112108223 | G | A | 0.338 | 0.024 | 1.47E-46 | 0.0005 | 205.276 |
| 18 | 11 | 45922456 | rs113933090 | A | G | 0.114 | 0.021 | 3.47E-08 | 0.0001 | 30.425 |
| 19 | 10 | 114736670 | rs114322470 | T | G | 0.233 | 0.023 | 3.35E-25 | 0.0003 | 107.565 |
| 20 | 8 | 118185733 | rs11558471 | A | G | 0.081 | 0.007 | 3.65E-33 | 0.0004 | 143.945 |
| 21 | 1 | 146740576 | rs11576360 | G | A | 0.051 | 0.008 | 2.26E-09 | 0.0001 | 35.74 |
| 22 | 15 | 67916400 | rs11631200 | A | G | 0.044 | 0.007 | 1.62E-11 | 0.0001 | 45.382 |
| 23 | 16 | 52207469 | rs11642255 | C | T | 0.09 | 0.015 | 4.68E-09 | 0.0001 | 34.317 |
| 24 | 8 | 10245788 | rs11774221 | A | G | 0.046 | 0.008 | 1.06E-08 | 0.0001 | 32.736 |
| 25 | 8 | 78101366 | rs118158020 | G | A | 0.094 | 0.017 | 4.64E-08 | 0.0001 | 29.861 |
| 26 | 9 | 34074476 | rs12001437 | C | T | 0.04 | 0.007 | 6.72E-09 | 0.0001 | 33.614 |
| 27 | 1 | 219793052 | rs12133396 | G | A | 0.043 | 0.008 | 1.51E-08 | 0.0001 | 32.036 |
| 28 | 7 | 72981883 | rs12531884 | A | C | 0.044 | 0.007 | 3.68E-11 | 0.0001 | 43.777 |
| 29 | 6 | 31108607 | rs1265089 | A | G | 0.044 | 0.008 | 1.63E-08 | 0.0001 | 31.896 |
| 30 | 10 | 112971811 | rs12780861 | C | A | 0.07 | 0.012 | 2.17E-09 | 0.0001 | 35.815 |
| 31 | 3 | 184748356 | rs13067541 | C | T | 0.069 | 0.013 | 3.02E-08 | 0.0001 | 30.697 |
| 32 | 1 | 203518873 | rs13303359 | A | C | 0.039 | 0.007 | 4.00E-09 | 0.0001 | 34.626 |
| 33 | 12 | 123913912 | rs141433349 | A | G | 0.125 | 0.019 | 3.17E-11 | 0.0001 | 44.07 |
| 34 | 22 | 30170655 | rs142682088 | G | A | 0.119 | 0.017 | 1.66E-12 | 0.0001 | 49.85 |
| 35 | 12 | 66242611 | rs149991550 | T | C | 0.193 | 0.022 | 3.89E-19 | 0.0002 | 79.923 |
| 36 | 10 | 114999579 | rs150654093 | A | G | 0.128 | 0.023 | 3.36E-08 | 0.0001 | 30.485 |
| 37 | 7 | 130453382 | rs1596972 | G | A | 0.051 | 0.007 | 6.11E-15 | 0.0002 | 60.865 |
| 38 | 3 | 12329783 | rs17036160 | C | T | 0.097 | 0.009 | 3.11E-28 | 0.0003 | 121.408 |
| 39 | 7 | 14898282 | rs17168486 | T | C | 0.053 | 0.008 | 6.59E-11 | 0.0001 | 42.638 |
| 40 | 1 | 51039300 | rs17383290 | A | G | 0.045 | 0.007 | 1.73E-09 | 0.0001 | 36.261 |
| 41 | 8 | 128711742 | rs17772814 | G | A | 0.078 | 0.011 | 3.20E-12 | 0.0001 | 48.562 |
| 42 | 18 | 21097460 | rs1788817 | A | G | 0.044 | 0.007 | 1.35E-11 | 0.0001 | 45.735 |
| 43 | 12 | 71535095 | rs1798090 | T | C | 0.039 | 0.007 | 2.49E-09 | 0.0001 | 35.55 |
| 44 | 6 | 131374647 | rs187642402 | C | T | 0.086 | 0.015 | 7.10E-09 | 0.0001 | 33.507 |
| 45 | 2 | 227103717 | rs2138157 | C | A | 0.077 | 0.007 | 1.63E-29 | 0.0003 | 127.263 |
| 46 | 11 | 2390172 | rs2521248 | T | C | 0.039 | 0.007 | 6.05E-09 | 0.0001 | 33.818 |
| 47 | 5 | 133887310 | rs2589408 | A | G | 0.04 | 0.007 | 1.86E-09 | 0.0001 | 36.114 |
| 48 | 15 | 77883802 | rs2682907 | G | A | 0.057 | 0.007 | 9.53E-18 | 0.0002 | 73.606 |
| 49 | 12 | 41866717 | rs2733269 | G | A | 0.037 | 0.007 | 1.86E-08 | 0.0001 | 31.635 |
| 50 | 10 | 71456857 | rs2812535 | A | G | 0.043 | 0.007 | 1.74E-09 | 0.0001 | 36.247 |
| 51 | 14 | 79943606 | rs28479795 | T | C | 0.052 | 0.008 | 8.39E-12 | 0.0001 | 46.673 |
| 52 | 9 | 139237902 | rs28624681 | C | T | 0.077 | 0.007 | 1.82E-26 | 0.0003 | 113.341 |
| 53 | 15 | 62309709 | rs28798715 | C | A | 0.068 | 0.01 | 4.54E-11 | 0.0001 | 43.364 |
| 54 | 16 | 81534790 | rs2925979 | T | C | 0.051 | 0.007 | 3.49E-13 | 0.0001 | 52.911 |
| 55 | 16 | 89376330 | rs3096301 | T | C | 0.042 | 0.007 | 1.24E-09 | 0.0001 | 36.906 |
| 56 | 9 | 81954187 | rs34029654 | G | A | 0.055 | 0.01 | 4.62E-08 | 0.0001 | 29.87 |
| 57 | 10 | 99214761 | rs34312169 | A | C | 0.05 | 0.009 | 1.06E-08 | 0.0001 | 32.721 |
| 58 | 1 | 229672955 | rs348330 | G | A | 0.048 | 0.007 | 4.26E-12 | 0.0001 | 48 |
| 59 | 7 | 74134911 | rs35005436 | C | T | 0.056 | 0.01 | 3.60E-08 | 0.0001 | 30.352 |
| 60 | 7 | 45460437 | rs35196956 | T | C | 0.114 | 0.018 | 1.30E-10 | 0.0001 | 41.302 |
| 61 | 17 | 66029590 | rs35229997 | G | T | 0.05 | 0.008 | 6.81E-11 | 0.0001 | 42.574 |
| 62 | 3 | 23455582 | rs35352848 | T | C | 0.069 | 0.007 | 5.32E-22 | 0.0002 | 92.966 |
| 63 | 5 | 102338811 | rs35658696 | G | A | 0.099 | 0.014 | 7.29E-13 | 0.0001 | 51.464 |
| 64 | 7 | 44231216 | rs3757840 | T | G | 0.056 | 0.007 | 1.16E-17 | 0.0002 | 73.216 |
| 65 | 4 | 85314016 | rs387955 | T | C | 0.1 | 0.014 | 5.43E-12 | 0.0001 | 47.525 |
| 66 | 3 | 186665645 | rs3887925 | T | C | 0.05 | 0.007 | 2.98E-14 | 0.0001 | 57.746 |
| 67 | 6 | 126964510 | rs4273712 | G | A | 0.046 | 0.007 | 3.65E-10 | 0.0001 | 39.29 |
| 68 | 18 | 40151204 | rs4289073 | G | T | 0.041 | 0.007 | 6.34E-10 | 0.0001 | 38.213 |
| 69 | 6 | 32075563 | rs429150 | C | T | 0.043 | 0.007 | 7.09E-11 | 0.0001 | 42.493 |
| 70 | 16 | 306243 | rs4374177 | A | G | 0.061 | 0.01 | 3.72E-09 | 0.0001 | 34.764 |
| 71 | 3 | 25383258 | rs4681047 | A | G | 0.041 | 0.007 | 2.86E-10 | 0.0001 | 39.768 |
| 72 | 19 | 7970635 | rs4804833 | A | G | 0.045 | 0.007 | 3.62E-11 | 0.0001 | 43.811 |
| 73 | 19 | 45416178 | rs483082 | G | T | 0.076 | 0.008 | 2.46E-22 | 0.0002 | 94.491 |
| 74 | 4 | 185726548 | rs4862423 | T | C | 0.046 | 0.007 | 1.04E-11 | 0.0001 | 46.253 |
| 75 | 13 | 33554405 | rs488321 | T | C | 0.08 | 0.011 | 1.17E-12 | 0.0001 | 50.54 |
| 76 | 11 | 128222116 | rs4937325 | T | C | 0.046 | 0.007 | 2.31E-11 | 0.0001 | 44.688 |
| 77 | 2 | 19680469 | rs55877252 | T | G | 0.073 | 0.013 | 4.23E-08 | 0.0001 | 30.04 |
| 78 | 3 | 137700873 | rs56901542 | A | G | 0.044 | 0.007 | 1.64E-09 | 0.0001 | 36.362 |
| 79 | 1 | 26606291 | rs61775082 | C | T | 0.056 | 0.01 | 7.43E-09 | 0.0001 | 33.419 |
| 80 | 1 | 39940499 | rs61779309 | C | T | 0.065 | 0.008 | 6.08E-15 | 0.0002 | 60.875 |
| 81 | 6 | 139826722 | rs618652 | T | G | 0.04 | 0.007 | 1.01E-09 | 0.0001 | 37.302 |
| 82 | 11 | 68658686 | rs61887147 | A | C | 0.047 | 0.008 | 2.44E-09 | 0.0001 | 35.587 |
| 83 | 18 | 52922007 | rs62092443 | T | C | 0.07 | 0.011 | 5.84E-10 | 0.0001 | 38.373 |
| 84 | 7 | 1953416 | rs62442903 | G | A | 0.041 | 0.007 | 2.81E-08 | 0.0001 | 30.832 |
| 85 | 7 | 102553366 | rs62484946 | C | T | 0.083 | 0.015 | 4.50E-08 | 0.0001 | 29.92 |
| 86 | 16 | 75245937 | rs66502159 | C | T | 0.114 | 0.011 | 6.54E-24 | 0.0003 | 101.675 |
| 87 | 18 | 57848651 | rs66922415 | G | A | 0.056 | 0.008 | 1.82E-11 | 0.0001 | 45.153 |
| 88 | 4 | 103872854 | rs6839635 | C | A | 0.043 | 0.007 | 5.87E-11 | 0.0001 | 42.865 |
| 89 | 4 | 153513937 | rs6850761 | A | C | 0.038 | 0.007 | 1.95E-08 | 0.0001 | 31.546 |
| 90 | 5 | 76427311 | rs6878122 | G | A | 0.06 | 0.008 | 7.98E-15 | 0.0002 | 60.339 |
| 91 | 6 | 137287702 | rs6918311 | A | G | 0.042 | 0.007 | 1.63E-10 | 0.0001 | 40.865 |
| 92 | 6 | 20703952 | rs6931514 | G | A | 0.118 | 0.007 | 9.09E-65 | 0.0007 | 288.795 |
| 93 | 7 | 99580371 | rs6944766 | G | A | 0.046 | 0.008 | 4.75E-08 | 0.0001 | 29.818 |
| 94 | 9 | 22137685 | rs7018475 | G | T | 0.093 | 0.007 | 3.22E-37 | 0.0004 | 162.499 |
| 95 | 11 | 72463435 | rs7109575 | G | A | 0.091 | 0.008 | 2.12E-31 | 0.0003 | 135.875 |
| 96 | 14 | 69751282 | rs7140574 | A | G | 0.04 | 0.007 | 1.10E-09 | 0.0001 | 37.138 |
| 97 | 14 | 24527672 | rs7146599 | A | G | 0.039 | 0.007 | 2.07E-09 | 0.0001 | 35.909 |
| 98 | 16 | 53821413 | rs7206629 | C | T | 0.117 | 0.007 | 6.92E-70 | 0.0008 | 312.288 |
| 99 | 14 | 38771529 | rs72683004 | C | A | 0.042 | 0.007 | 8.83E-09 | 0.0001 | 33.082 |
| 100 | 10 | 70981553 | rs72812178 | T | G | 0.077 | 0.009 | 2.66E-16 | 0.0002 | 67.043 |
| 101 | 2 | 58712420 | rs72813935 | T | C | 0.084 | 0.013 | 2.57E-10 | 0.0001 | 39.978 |
| 102 | 12 | 4362909 | rs73040004 | C | T | 0.071 | 0.008 | 1.98E-19 | 0.0002 | 81.26 |
| 103 | 12 | 97504719 | rs7306319 | A | G | 0.045 | 0.008 | 4.74E-09 | 0.0001 | 34.295 |
| 104 | 12 | 121824009 | rs73224247 | T | C | 0.068 | 0.011 | 1.03E-09 | 0.0001 | 37.26 |
| 105 | 14 | 101255172 | rs73347525 | A | G | 0.055 | 0.008 | 6.23E-12 | 0.0001 | 47.257 |
| 106 | 12 | 97851002 | rs74628648 | C | T | 0.077 | 0.012 | 3.83E-10 | 0.0001 | 39.199 |
| 107 | 2 | 166733738 | rs749773 | C | T | 0.04 | 0.007 | 3.30E-08 | 0.0001 | 30.525 |
| 108 | 2 | 620297 | rs7563362 | G | A | 0.065 | 0.009 | 5.91E-12 | 0.0001 | 47.359 |
| 109 | 3 | 12296751 | rs7631359 | T | G | 0.059 | 0.011 | 2.29E-08 | 0.0001 | 31.232 |
| 110 | 4 | 96145196 | rs7661876 | G | T | 0.041 | 0.007 | 4.62E-10 | 0.0001 | 38.832 |
| 111 | 14 | 101250258 | rs77789961 | T | C | 0.085 | 0.016 | 3.86E-08 | 0.0001 | 30.216 |
| 112 | 7 | 150536681 | rs7788808 | C | T | 0.051 | 0.008 | 2.43E-11 | 0.0001 | 44.589 |
| 113 | 8 | 95937502 | rs7845219 | T | C | 0.043 | 0.007 | 5.78E-11 | 0.0001 | 42.892 |
| 114 | 5 | 14690667 | rs78486128 | A | G | 0.065 | 0.011 | 1.06E-09 | 0.0001 | 37.209 |
| 115 | 11 | 983626 | rs78721871 | A | G | 0.116 | 0.017 | 2.07E-11 | 0.0001 | 44.901 |
| 116 | 7 | 101959831 | rs79356898 | A | G | 0.098 | 0.014 | 3.05E-12 | 0.0001 | 48.653 |
| 117 | 15 | 52533066 | rs79400013 | G | A | 0.156 | 0.025 | 3.74E-10 | 0.0001 | 39.243 |
| 118 | 2 | 43469615 | rs80323638 | G | A | 0.144 | 0.016 | 5.67E-20 | 0.0002 | 83.73 |
| 119 | 17 | 3988451 | rs8071043 | C | T | 0.062 | 0.007 | 3.28E-18 | 0.0002 | 75.715 |
| 120 | 17 | 7440584 | rs8073177 | T | C | 0.052 | 0.008 | 4.34E-12 | 0.0001 | 47.963 |
| 121 | 19 | 19393714 | rs8100204 | A | G | 0.072 | 0.009 | 1.26E-15 | 0.0002 | 63.969 |
| 122 | 11 | 2798305 | rs81204 | T | C | 0.041 | 0.008 | 4.89E-08 | 0.0001 | 29.762 |
| 123 | 20 | 62711459 | rs8126001 | C | T | 0.041 | 0.007 | 6.32E-10 | 0.0001 | 38.219 |
| 124 | 7 | 28232457 | rs849327 | A | G | 0.067 | 0.007 | 3.52E-22 | 0.0002 | 93.785 |
| 125 | 11 | 93255014 | rs897558 | G | A | 0.041 | 0.007 | 3.89E-10 | 0.0001 | 39.167 |
| 126 | 6 | 22021373 | rs9350408 | C | T | 0.04 | 0.007 | 1.03E-09 | 0.0001 | 37.265 |
| 127 | 6 | 7232186 | rs9505086 | C | T | 0.057 | 0.007 | 7.15E-18 | 0.0002 | 74.174 |
| 128 | 5 | 55861894 | rs9687846 | A | G | 0.058 | 0.01 | 1.40E-09 | 0.0001 | 36.665 |
| 129 | 17 | 47079416 | rs9909861 | C | A | 0.045 | 0.007 | 1.58E-10 | 0.0001 | 40.927 |
| 130 | 17 | 36105897 | rs9913260 | A | G | 0.049 | 0.008 | 6.27E-09 | 0.0001 | 33.748 |

**Supplementary Table 7.** The summary information for instrumental variables in obesity and FST.

| **Sort** | **chr** | **pos** | **SNP** | **effect_allele** | **other_allele** | **beta** | **se** | **pval** | ***R^2^*** | ***F*** |
| --- | --- | --- | --- | --- | --- | --- | --- | --- | --- | --- |
| 1 | 12 | 103371966 | rs10860990 | T | C | 0.057 | 1.00E-02 | 6.31E-09 | 8.20E-05 | 33.736 |
| 2 | 10 | 86270684 | rs10887571 | T | C | 0.054 | 1.00E-02 | 1.88E-08 | 7.70E-05 | 31.61 |
| 3 | 2 | 160428726 | rs10929985 | T | C | 0.053 | 1.00E-02 | 4.31E-08 | 7.30E-05 | 30.004 |
| 4 | 11 | 27662970 | rs11030104 | A | G | 0.104 | 1.30E-02 | 2.35E-15 | 1.52E-04 | 62.745 |
| 5 | 18 | 42554092 | rs11082279 | G | A | 0.057 | 1.00E-02 | 5.42E-09 | 8.30E-05 | 34.032 |
| 6 | 12 | 72432850 | rs11179203 | A | G | 0.07 | 1.30E-02 | 4.52E-08 | 7.30E-05 | 29.912 |
| 7 | 15 | 67731471 | rs12050481 | C | T | 0.058 | 1.00E-02 | 6.64E-09 | 8.20E-05 | 33.637 |
| 8 | 4 | 45481104 | rs12511535 | C | T | 0.055 | 1.00E-02 | 2.72E-08 | 7.50E-05 | 30.897 |
| 9 | 3 | 35034339 | rs12639495 | C | T | 0.085 | 1.20E-02 | 1.06E-11 | 1.12E-04 | 46.214 |
| 10 | 12 | 17621984 | rs12831874 | G | T | 0.077 | 1.40E-02 | 1.80E-08 | 7.70E-05 | 31.7 |
| 11 | 2 | 26706414 | rs13394970 | T | G | 0.053 | 1.00E-02 | 3.26E-08 | 7.40E-05 | 30.549 |
| 12 | 13 | 58101165 | rs1445594 | A | G | 0.07 | 1.00E-02 | 8.55E-12 | 1.13E-04 | 46.635 |
| 13 | 15 | 46267299 | rs1594904 | G | A | 0.057 | 1.00E-02 | 1.73E-08 | 7.70E-05 | 31.771 |
| 14 | 1 | 109604699 | rs17024258 | T | C | 0.118 | 2.00E-02 | 1.74E-09 | 8.80E-05 | 36.24 |
| 15 | 18 | 60181298 | rs2168711 | C | T | 0.103 | 1.20E-02 | 1.20E-17 | 1.78E-04 | 73.157 |
| 16 | 19 | 45677156 | rs34783010 | G | T | 0.078 | 1.10E-02 | 1.51E-12 | 1.21E-04 | 50.037 |
| 17 | 6 | 143625455 | rs35296418 | G | A | 0.072 | 1.20E-02 | 1.73E-09 | 8.80E-05 | 36.26 |
| 18 | 1 | 151254895 | rs3811405 | G | A | 0.059 | 1.10E-02 | 3.37E-08 | 7.40E-05 | 30.484 |
| 19 | 19 | 18364865 | rs4072287 | A | C | 0.062 | 1.00E-02 | 2.88E-10 | 9.60E-05 | 39.753 |
| 20 | 20 | 59032308 | rs45551238 | C | T | 0.143 | 2.30E-02 | 5.70E-10 | 9.30E-05 | 38.422 |
| 21 | 1 | 177925456 | rs509325 | G | T | 0.081 | 1.20E-02 | 3.61E-11 | 1.06E-04 | 43.815 |
| 22 | 5 | 168100306 | rs59563471 | A | G | 0.065 | 1.10E-02 | 5.52E-09 | 8.30E-05 | 33.997 |
| 23 | 7 | 76970989 | rs62473704 | C | A | 0.106 | 1.20E-02 | 3.28E-19 | 1.95E-04 | 80.263 |
| 24 | 1 | 194783257 | rs66466969 | A | C | 0.055 | 1.00E-02 | 4.94E-08 | 7.20E-05 | 29.742 |
| 25 | 2 | 136664268 | rs6726297 | G | A | 0.066 | 1.10E-02 | 2.47E-09 | 8.60E-05 | 35.563 |
| 26 | 2 | 628749 | rs6739303 | T | C | 0.129 | 1.30E-02 | 1.31E-22 | 2.32E-04 | 95.742 |
| 27 | 2 | 24888093 | rs6749170 | G | A | 0.053 | 1.00E-02 | 3.78E-08 | 7.30E-05 | 30.258 |
| 28 | 14 | 32839824 | rs7140259 | G | T | 0.055 | 1.00E-02 | 8.58E-09 | 8.00E-05 | 33.139 |
| 29 | 18 | 1850770 | rs7226371 | G | A | 0.078 | 1.20E-02 | 3.57E-10 | 9.50E-05 | 39.334 |
| 30 | 13 | 54125449 | rs76503812 | A | G | 0.104 | 1.90E-02 | 4.12E-08 | 7.30E-05 | 30.091 |

**Supplementary Table 8.** The summary information for instrumental variables in osteoporosis and FST.

| **Sort** | **chr** | **pos** | **SNP** | **effect_allele** | **other_allele** | **beta** | **se** | **pval** | ***R^2^*** | ***F*** |
| --- | --- | --- | --- | --- | --- | --- | --- | --- | --- | --- |
| 1 | 7 | 30956489 | rs10276670 | G | A | 0.118 | 0.021 | 4.43E-08 | 7.50E-05 | 29.949 |
| 2 | 12 | 49385679 | rs10875906 | C | T | 0.106 | 0.017 | 1.11E-09 | 9.30E-05 | 37.126 |
| 3 | 21 | 40350120 | rs11088458 | G | A | 0.097 | 0.017 | 1.69E-08 | 7.97E-05 | 31.821 |
| 4 | 6 | 127425630 | rs1963689 | T | C | 0.106 | 0.019 | 4.28E-08 | 7.52E-05 | 30.017 |
| 5 | 6 | 32659332 | rs5002706 | A | G | 0.129 | 0.023 | 4.11E-08 | 7.54E-05 | 30.099 |
| 6 | 17 | 41808374 | rs80107551 | C | T | 0.150 | 0.026 | 8.03E-09 | 8.34E-05 | 33.268 |
