# Supplementary material for: Follistatin levels and endocrine disorders: A two-sample Mendelian randomization study
Source: Medicine (Baltimore). 2025 Oct 31;104(44):e45566. doi: 10.1097/MD.0000000000045566 (PMC12582763; doi:10.1097/MD.0000000000045566)
Supplement: Supplementary file 2 [file medi-104-e45566-s002.pdf]

Supplementary Figure 1. The “leave-one-out” plots of the causality between FST levels and PCOS.

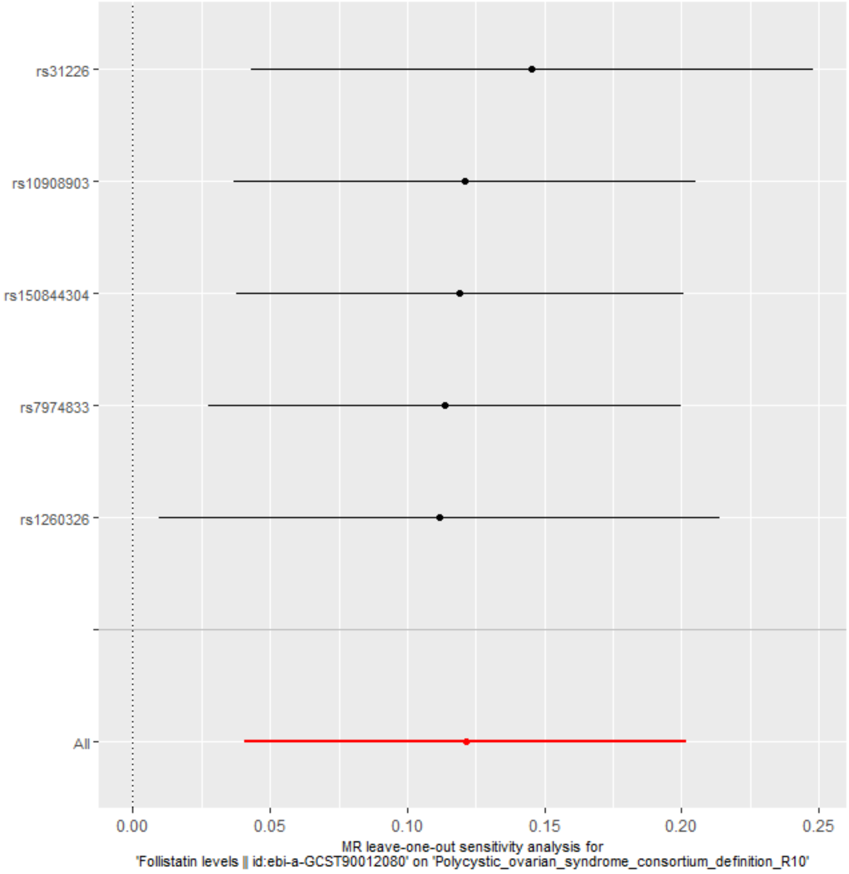

Supplementary Figure 2. The “leave-one-out” plots of the causality between FST levels and T2DM.

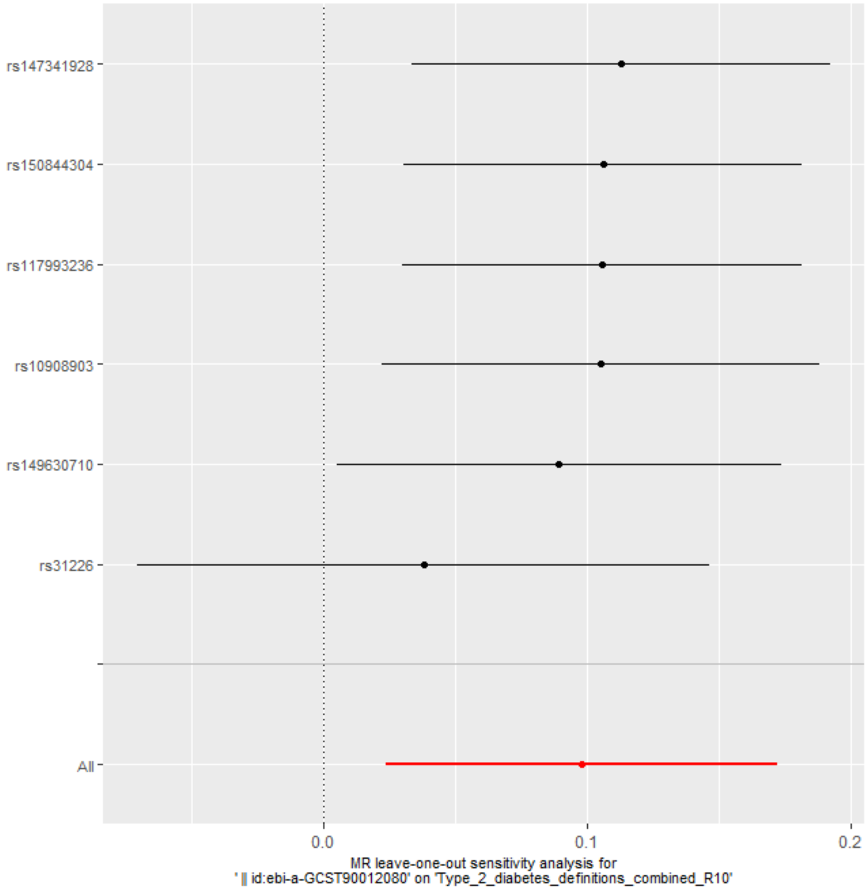

Supplementary Figure 3. The "leave-one-out" plots of the causality between FST levels and

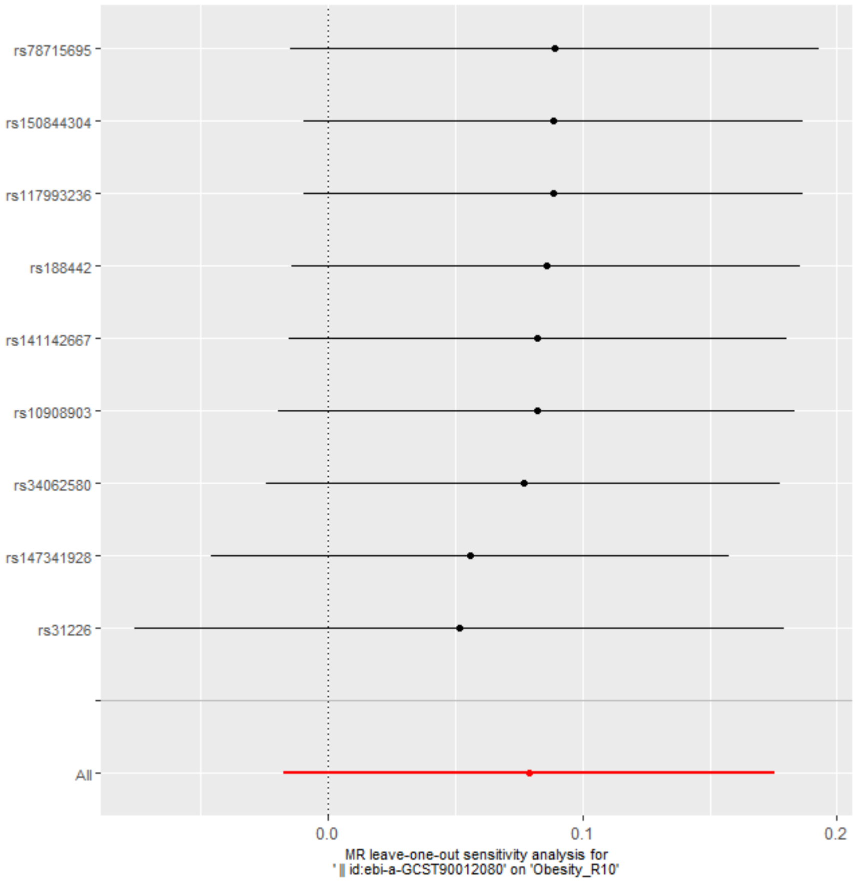

obesity.

Supplementary Figure 4. The “leave-one-out” plots of the causality between FST levels and

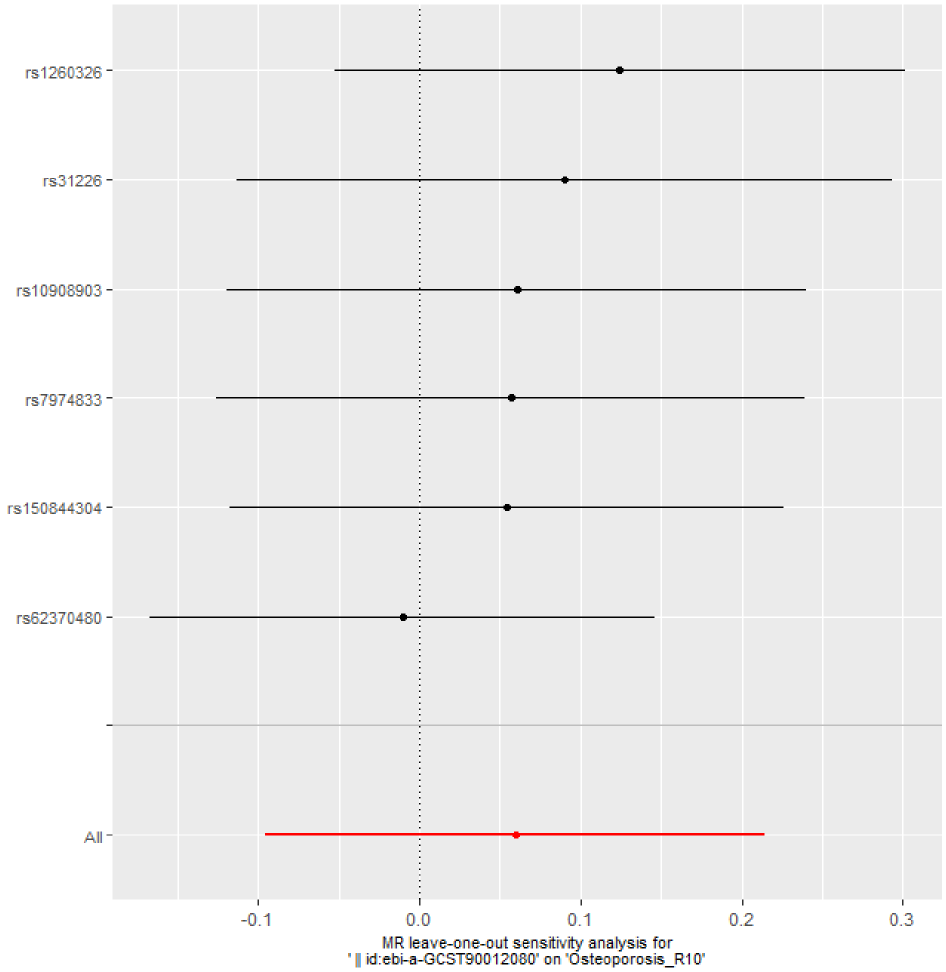

osteoporosis.

Supplementary Figure 5. The “leave-one-out” plots of the causality between PCOS and FST levels.

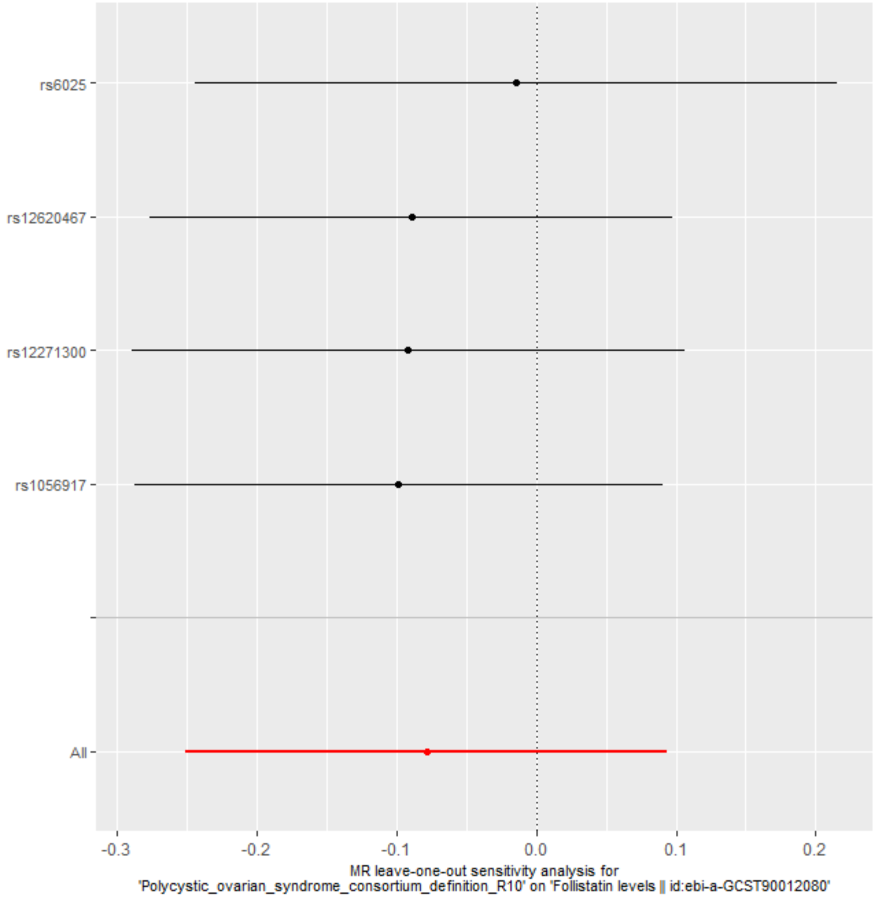

Supplementary Figure 6. The “leave-one-out” plots of the causality between T2DM and FST levels.

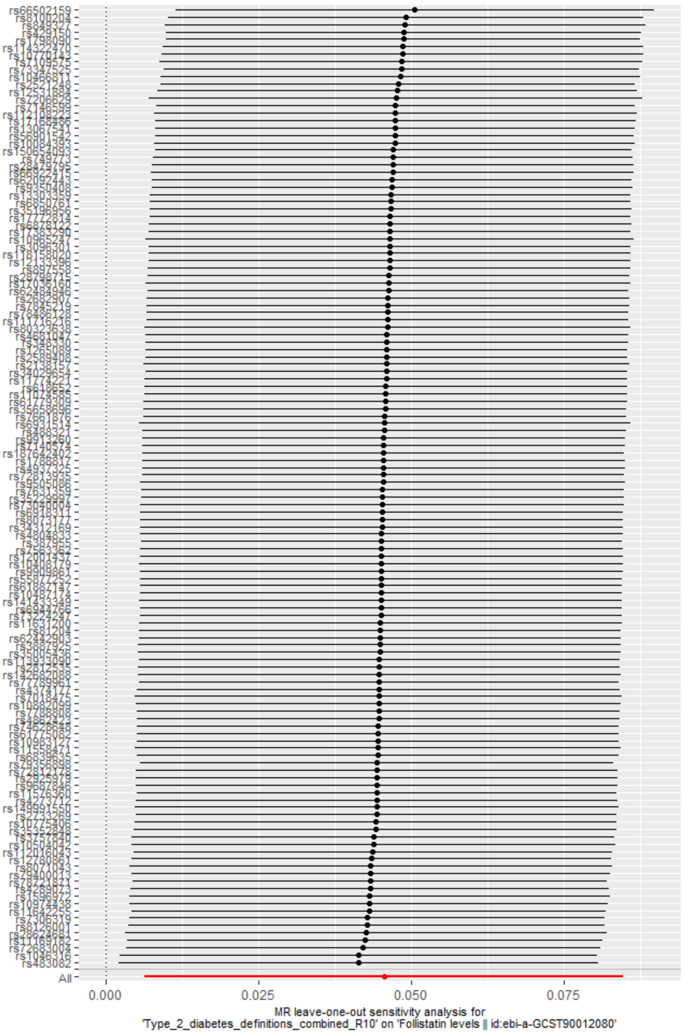

Supplementary Figure 7. The “leave-one-out” plots of the causality between obesity and FST levels.

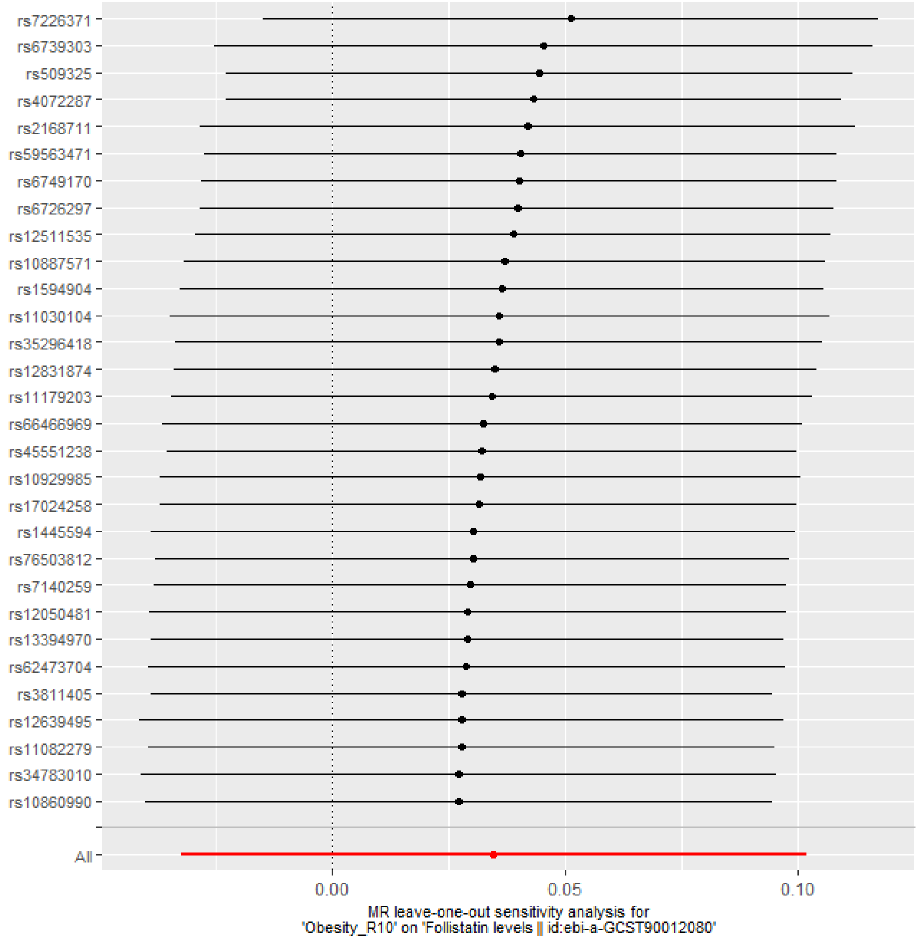

Supplementary Figure 8. The "leave-one-out" plots of the causality between osteoporosis and FST

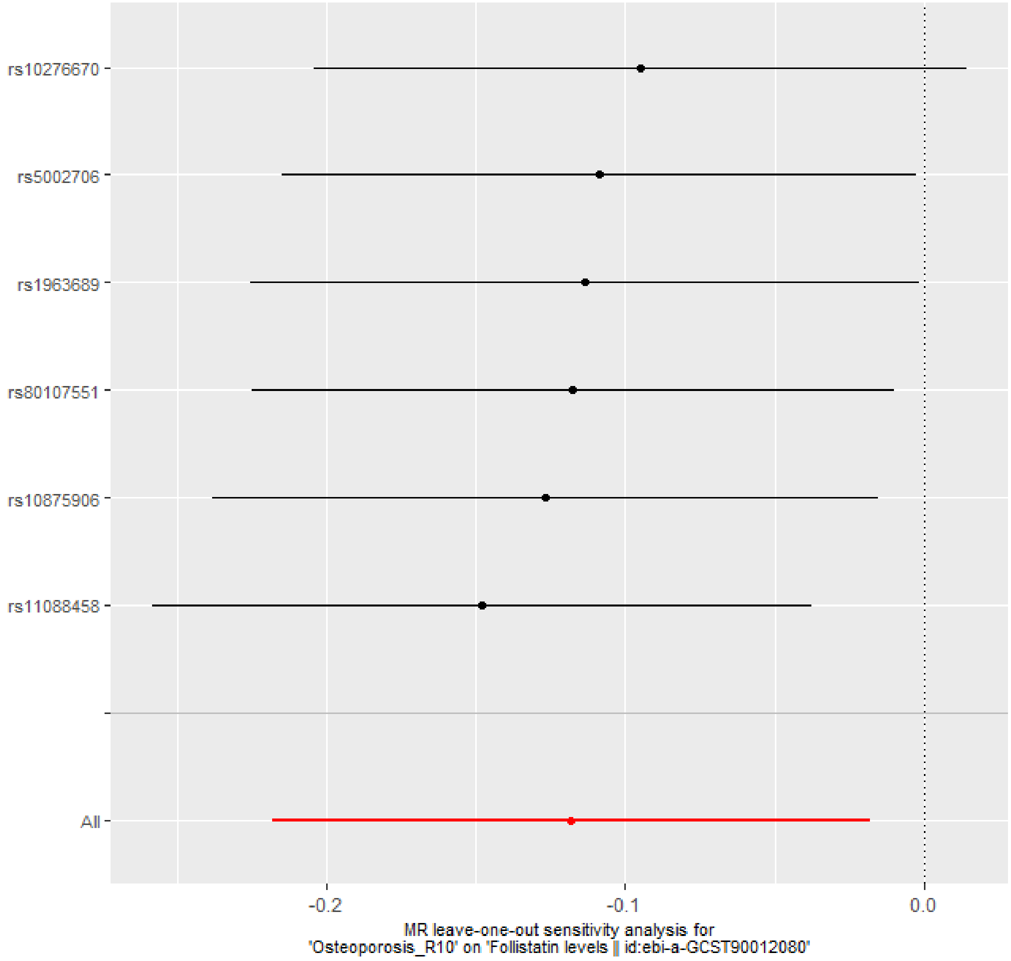

levels.
